# First Signs at Admission: Prognostic Value of Initial Proinflammatory Markers for Sepsis and Mortality in ICU Patients

**DOI:** 10.3390/pathogens14090907

**Published:** 2025-09-10

**Authors:** Fatma Aktaş, Yasemin Zer, Gülseren Elay, Mehmet Erinmez

**Affiliations:** 1 Department of Medical Microbiology, Ersin Arslan Education and Research Hospital, Gaziantep 27090, Türkiye; dr.fatmaktas@gmail.com; 2 Department of Medical Microbiology, Faculty of Medicine, Gaziantep University, Gaziantep 27310, Türkiye; yaseminzer@hotmail.com; 3 Department of Internal Medicine, Faculty of Medicine, Gaziantep University, Gaziantep 27310, Türkiye; gulserenelay56@gmail.com

**Keywords:** sepsis, bacteremia, biomarker, proinflammatory markers

## Abstract

Sepsis is a life-threatening condition and due to its non-specific symptoms, diagnosing sepsis and determining its severity remains difficult. Delays in recognizing sepsis can significantly increase mortality despite advances in antimicrobial therapy and resuscitation procedures. Biomarkers can help detect the presence and severity of sepsis, distinguish between types of infections, and evaluate treatment response. This prospective study aims to determine whether biomarker levels measured at the time of intensive care unit (ICU) admission can assist in the early prediction, diagnosis, and prognosis of sepsis and bacteremia. Blood samples were collected from 132 ICU patients upon admission and analyzed for CRP, IL-6, PCT, SAA, and endotoxin levels. Patients were monitored for sepsis development, blood culture results, and mortality. IL-6 levels demonstrated a significant association with prognosis and identified as an independent risk factor. CRP and PCT levels exhibit a significant effect on the development of sepsis in both univariate and multivariate analyses. Also, our study demonstrated that the presence of bacteremia in the initial blood sample taken from intensive care patients holds significant diagnostic and prognostic value even without waiting for species-level identification when combined with markers such as PCT, CRP, and IL-6.

## 1. Introduction

The septic response is a very intricate series of events that includes humoral and cellular reactions, circulatory abnormalities, and inflammatory and anti-inflammatory mechanisms [1]. It is challenging to accurately identify sepsis and determine its severity due to the incredibly varied and non-specific characteristics of its symptoms [2]. For critically ill patients, sepsis continues to be a leading cause of death despite advances in antimicrobial therapy and resuscitation procedures [3]. However, early identification and evaluation of the severity of sepsis are essential since they raise the possibility of starting prompt, directed treatment [4]. Delays in initiating effective antimicrobial therapy have been associated with a 7–10% increase in sepsis-related mortality per hour, primarily due to failure in timely recognition, which remains a common diagnostic challenge [5].

Although the terms sepsis and bloodstream infection (BSI) are frequently used synonymously in non-medical manuscripts, they refer to distinct concepts [6]. BSI is characterized by a pathogenic organism in the bloodstream that exhibits systemic signs of infection. It might be primary, meaning its origin is unknown, or secondary to an identified cause [7]. When the bloodstream infection is caused by bacteria, it is referred to as bacteremia [6]. The association between sepsis and bacteremia is also somewhat not complete. Sepsis is not always the outcome of bacteremia; in many cases, the infection is controlled before organ damage and a dysregulated host response arise [6]. Furthermore, not all cases of sepsis are due to bloodstream infections. In fact, bloodstream infections cause only 25–30% of sepsis cases [8]. In similar fashion, BSI diagnosis might be quite intricate. The gold standard for identifying the causative microorganism in sepsis and BSI remains culture-based methods [7]. Yet, growth in a blood culture does not necessarily indicate an infection, as contamination is a potential confounding factor [9]. Furthermore, growth may not be seen in blood cultures from certain sepsis patients. The clinical condition known as culture-negative sepsis may be caused by the host's reaction to certain bacterial components, such as endotoxins, in the circulatory system, or it may be associated with the start of antibiotic treatment prior to blood cultures being obtained or another important factor is that certain pathogenic bacteria are not readily culturable [10,11]. Additionally, the blood culture growth time requirements impose a schedule that is extremely incompatible with the urgency of sepsis [7,9,11]. Given its complex pathophysiology, it is unsurprising that no single laboratory test can definitively diagnose sepsis. Therefore, researchers have investigated a number of biomarkers in an effort to increase accuracy of sepsis diagnosis [7]. 

Biomarkers, defined as laboratory variables or indicators, are used in order to diagnose a disease and objectively assess the response to treatment [12]. In the early 1990s, researchers reported that serum concentrations of a substance immunologically identical to procalcitonin are elevated during septic conditions and appear to correlate with the severity of microbial invasion [13]. This was subsequently complemented identifying interleukin 6 as a prognostic indicator of outcome in severe intra-abdominal sepsis [14]. Over 250 sepsis biomarkers have been identified in recent years, with ongoing discoveries [15]. Biomarkers may be crucial for managing sepsis as they may suggest whether or not sepsis is present and how severe it is [16]. In addition, biomarkers are able to differentiate between bacterial, viral, and fungal infections, as well as between systemic sepsis and local infection [17]. Biomarker evaluation studies are often limited by factors such as selected or heterogeneous patient populations, variability in reference standards, and bias in the choice of the gold standard for defining sepsis, which can affect the reliability and generalizability of their findings [15,18]. Despite the lack of a definitive biomarker for sepsis, a combination of biomarkers is crucial for assessing diagnosis, staging, prognosis, and intervention results [7,19]. This study investigates whether biomarker levels measured upon intensive care unit (ICU) admission can aid in the prediction, diagnosis, and prognosis of suspected sepsis and bacteremia, with the goal of enhancing early recognition and management.

## 2. Materials and Methods

### 2.1. Patient Characteristics

In this prospective study, patients were recruited from the ICU of the Department of Internal Medicine between September 2023 and April 2024.

Inclusion criteria:

1. Age ≥ 18 years;
2. Admitted to the ICU with suspected bacteremia (based on clinical presentation and treating physician’s assessment);
3. First blood sampling performed within 24 h of ICU admission;
4. Provision of written informed consent by the patient or a legal representative.

Exclusion criteria:

1. Known diagnosis of sepsis or bacteremia at the time of ICU admission;
2. Previous enrollment in the study;
3. Receipt of antibiotics for more than 48 h before ICU admission;
4. Refusal or inability to provide informed consent.

A total of 132 patients were included. The sample size was determined a priori by power analysis, which indicated that this number of participants was sufficient to detect clinically meaningful differences in sepsis outcomes with adequate statistical power. For each eligible patient, an additional 8–10 cc of venous blood was collected at the time of the first routine blood sampling. Patients were prospectively monitored for blood culture results, the development of sepsis, and mortality. Blood samples were allowed to clot at room temperature for 30 minutes, centrifuged at 3000 rpm for 15 minutes, divided into three aliquots, and stored at −20 °C until analysis.

### 2.2. Microbiological Procedures

Blood cultures were studied with the BACTEC FX System (Beckton Dickinson, Franklin Lakes, NJ, USA). Culture samples that exhibited growth during the five-day incubation period were plated on 5% sheep blood agar (BD, Heidelberg, Germany) and Eosin Methylene Blue (EMB) (BD, Heidelberg, Germany) agar, then incubated at 37 °C for 24 to 48 h. Identification of the growing bacteria was performed using the BD Phoenix (Beckton Dickinson, Franklin Lakes, NJ, USA) automated Bacteria Identification System and conventional methods. Two distinct study groups were established based on blood culture growth results: Group I (negative) and Group II (positive). Coagulase-Negative Staphylococci (CoNS) isolates were considered true bacteremia only if recovered from two or more separate blood culture sets drawn at different times. Single positive cultures without clinical evidence of infection (fever, hemodynamic instability, or elevated inflammatory markers) were regarded as likely contaminants and excluded from the analysis.

### 2.3. Bioamarker Evaluation

Serum samples, stored at −20 ℃ during the study period, were brought to room temperature, and the following biomarkers were analyzed prospectively: C-Reactive protein (CRP), Interleukin-6 (IL-6), Procalcitonin (PCT), serum amyloid A (SAA) and Serum Endotoxin level. CRP, PCT, IL-6, and SAA levels were measured using chemiluminescence immunoassay (CLIA) on the Maglumi X6 (Snibe Diagnostic, Shenzhen, China), while endotoxin levels were quantitatively assessed using the micro-scale Enzyme-linked Immunosorbent Assay (micro-ELISA; BT Lab, Shanghai, China) technique. According to the manufacturer's recommendations, the reference values were as follows: IL-6 (0–7 pg/mL), CRP (0–700 ng/mL), SAA (0–10 µg/mL), and PCT (0–0.5 ng/mL). For the determination of endotoxin, quantitative measurements were made using standards at concentrations of 100, 50, 25, 12.5, and 6.25 pg/mL. Endotoxin levels were measured solely in Group I, serving as the control group, and in Group II patients with bacteremia caused by Gram-negative pathogens.

### 2.4. Sepsis Definition 

Study participants were categorized into sepsis and non-sepsis groups based on established diagnostic criteria. Sepsis is identified as a systemic inflammatory response syndrome (SIRS) triggered by an infection and it is diagnosed when at least two of the following four physiological criteria are met [20]:

1. Body temperature higher than 38 °C or lower than 36 °C;
2. Heart rate exceeding 90 beats per minute;
3. Respiratory rate greater than 20 breaths per minute or arterial carbon dioxide tension below 32 mm Hg (4.3 kPa);
4. White blood cell counts above 11 or below 4 (×10^9^/L), or the presence of more than 10% immature (band) forms.

A diagnosis of sepsis is made when these SIRS criteria are met in conjunction with one of the following indicators of infection [21]:

1. A suspected infection being investigated through blood cultures and/or empirically treated with antibiotics;
2. A clinically evident infection;
3. A microbiologically confirmed infection.

In the early stages of sepsis, organ dysfunction, which is the essential feature defining sepsis under Sepsis 3, may not be readily apparent or easily measurable [22]. Therefore, SIRS based criteria were employed to identify patients at risk during this initial phase.

### 2.5. Statistical Method

Data analysis was performed using SPSS (Statistical Package for Social Sciences for Windows, Release ver. 29.0) software. Descriptive statistics, including mean, standard deviation, and percentage distributions, were reported. Kolmogorov–Smirnov and Shapiro–Wilk tests were used to determine whether the data were normally distributed. Comparisons of groups with were made with the independent samples t-test, chi-square test, Kruskal–Wallis-H test and Mann–Whitney U Test to determine specific group differences. Receiver operating characteristic (ROC) curve analysis was used to calculate optimal diagnostic cut-off values, which were determined using the Youden index. The statistical significance of the area under the ROC curve (AUC) was tested against the null hypothesis (AUC = 0.5) using the nonparametric method of Hanley and McNeil, equivalent to the Mann–Whitney U statistic, as implemented in SPSS. To investigate factors associated with sepsis, univariate and multivariate logistic regression models were constructed. Multivariate logistic regression analyses were performed using the Enter (forced entry) method, including clinically relevant variables identified from univariate analyses. To account for multiple testing in the univariate analyses, Bonferroni correction was applied, and adjusted *p*-values are presented in the Supplementary Tables S1–S4. Logistic regression analyses were also performed to identify predictors of patient outcomes (death or discharge). Model explanatory power was evaluated using appropriate statistical criteria, including R^2^. Spearman’s correlation analysis was used to assess relationships between continuous variables. A *p*-value < 0.05 was considered statistically significant.

## 3. Results

### 3.1. Patient Characteristics

A total of 132 patients were included in the study and classified based on their blood culture results. Of these, 63 patients (47.7%) were assigned to Group I (culture negative), 69 patients (52.3%) to Group II (positive). The cohort consisted of 80 males (60.6%) and 52 females (39.3%). The age of male patients ranged from 18 to 89 years, while the female patients’ ages ranged from 18 to 97 years. Detailed demographic, clinical, and laboratory findings of patients are given in Table 1.

### 3.2. Microbiological Findings

In Group II, among the 69 patients, Gram-negative bacteria were isolated in 32 patients (46.3%), Gram-positive bacteria in 29 patients (42%), and yeasts in 8 patients (11.5%). The details of the isolated pathogens are presented in Table 2. No statistically significant differences were observed between patients with positive and negative blood cultures regarding age, sex, underlying pathology, prognosis, time to death, or length of stay in the intensive care unit. In our cohort, the median time to blood culture positivity was 20.6 h (IQR 17.5–23.1 h), as recorded automatically by the blood culture monitoring system. While this demonstrates the potential for rapid detection, it should be noted that continuous 24-h monitoring of the device is not always feasible in routine practice, which may slightly delay recognition of positivity in real-world settings.

### 3.3. Biomarker Analysis

#### 3.3.1. IL-6

In our study group, IL-6 levels ranged from 1 to 5000 pg/mL, with a median value of 120 pg/ml and an interquartile range (IQR) of 335.75. No statistically significant difference was observed in IL-6 levels between patients with positive and negative blood cultures (*p* = 0.202). Additionally, no statistically significant difference was observed in IL-6 levels between patients diagnosed with sepsis and those without a sepsis diagnosis (*p* = 0.351). Furthermore, IL-6 levels did not exhibit a significant effect on the development of sepsis in either univariate or multivariate analyses (Table 3). Based on the ROC analysis, a cut-off value of 808 pg/mL was identified and IL-6 demonstrated a sensitivity of 63% and a specificity of 62% for sepsis prediction (*p* = 0.44) (Table 4). However, when the relationship between prognosis and IL-6 levels was examined, IL-6 levels were found to be higher in deceased patients compared to those who were discharged, and this difference was statistically significant (*p* = 0.013). IL-6 levels demonstrated a significant association with prognosis, with an odds ratio of 4.48 and a *p*-value of 0.001 in the univariate analysis (Table 5). In the multivariate analysis, IL-6 levels were identified as an independent risk factor, showing an odds ratio of 4.123 and a *p*-value of 0.001 (Table 5). In prognosis prediction, IL-6 levels alone demonstrated strong discriminative performance. A cut-off value of 639 pg/mL was identified, at which IL-6 achieved 74% sensitivity, 73% specificity, and a high diagnostic accuracy with an area under the curve (AUC) of 0.78 (*p* < 0.001) (Table 6). In the correlation analysis, IL-6 levels were found to have a positive and moderate correlation with CRP and PCT, with correlation coefficients of 0.49 and 0.65, respectively.

#### 3.3.2. CRP

In our study group, CRP levels ranged from 0.13 to 100,000 ng/mL, with a median value of 100,000 ng/mL and IQR of 55,990. No statistically significant difference was observed in CRP levels between patients with positive and negative blood cultures (*p* = 0.270). However, a statistically significant difference was observed in CRP levels between patients diagnosed with sepsis and those without a sepsis diagnosis (*p* = 0.023). CRP levels exhibit a significant effect on the development of sepsis in both univariate and multivariate analyses (Table 3). A cut-off value of 831 ng/mL was identified and CRP demonstrated a sensitivity of 82% and a specificity of 75% for sepsis prediction (*p* = 0.006) (Table 4). Furthermore, when the relationship between prognosis and CRP levels was examined, CRP levels were found to be higher in deceased patients compared to those who were discharged, and this difference was statistically significant (*p* = 0.001). CRP levels did not show a statistically significant effect on prognosis in either univariate or multivariate analyses (Table 5). In prognosis prediction, a cut-off value of 817 ng/mL was identified, at which CRP achieved 68% sensitivity and 65% specificity (*p* = 0.001) (Table 6). In the correlation analysis, CRP levels were found to have a positive and moderate correlation with PCT, with correlation coefficient of 0.51.

#### 3.3.3. PCT

In our study group, PCT levels ranged from 0.06 to 100 ng/mL, with a median value of 0.5 ng/mL and IQR of 0.4. A statistically significant difference was observed in PCT levels between patients with positive and negative blood cultures (*p* = 0.038). Also, a statistically significant difference was observed in PCT levels between patients diagnosed with sepsis and those without a sepsis diagnosis (*p* = 0.015). PCT levels exhibit a significant effect on the development of sepsis in both univariate and multivariate analyses (Table 3). A cut-off value of 1.8 ng/mL was identified and PCT demonstrated a sensitivity of 78% and a specificity of 70% for sepsis prediction (*p* = 0.001) (Table 4). Furthermore, when the relationship between prognosis and PCT levels was examined, no statistically significant difference was observed in PCT levels of deceased patients compared to those who were discharged (*p* = 0.399). PCT levels did not show a statistically significant effect on prognosis in either univariate or multivariate analyses (Table 5). 

#### 3.3.4. SAA

In our study group, SAA levels ranged from 0.15 to 300 µg/mL, with a median value of 300 µg/mL and IQR of 62.4. No statistically significant difference was observed in SAA levels between patients with positive and negative blood cultures (*p* = 0.414). Also, no statistically significant difference was observed in SAA levels between patients diagnosed with sepsis and those without a sepsis diagnosis (*p* = 0.458). SAA levels did not exhibit a significant effect on the development of sepsis in either univariate or multivariate analyses (Table 3). Furthermore, when the relationship between prognosis and SAA levels was examined, no statistically significant difference was observed in SAA levels of deceased patients compared to those who were discharged (*p* = 0.103). SAA levels did not show a statistically significant effect on prognosis in either univariate or multivariate analyses (Table 5). 

#### 3.3.5. Endotoxin

In our study group, endotoxin levels ranged from 0 to 180.4 pg/mL, with a median value of 46.3 pg/mL and IQR of 39.83. A statistically significant difference was observed in endotoxin levels between patients with positive and negative blood cultures (*p* = 0.001). However, no statistically significant difference was observed in endotoxin levels between patients diagnosed with sepsis and those without a sepsis diagnosis (*p* = 0.238). Endotoxin levels did not exhibit a significant effect on the development of sepsis in either univariate or multivariate analyses (Table 3). A cut-off value of 63.6 pg/mL was identified and endotoxin demonstrated a sensitivity of 41% and a specificity of 41% for sepsis prediction (*p* = 0.122) (Table 4). Furthermore, when the relationship between prognosis and endotoxin levels was examined, no statistically significant difference was observed in endotoxin levels of deceased patients compared to those who were discharged (*p* = 0.344). Endotoxin levels did not show a statistically significant effect on prognosis in either univariate or multivariate analyses (Table 5). In prognosis prediction, a cut-off value of 513 pg/mL was identified, at which endotoxin achieved 53% sensitivity and 41% specificity (*p* = 0.187) (Table 6). Endotoxin levels were measured only in patients with Gram-negative bacteremia, as endotoxin is specific to Gram-negative bacterial cell walls. Consequently, the potential contribution of Gram-negative co-infections in patients with Gram-positive bacteremia cannot be fully excluded. This limits the generalizability of endotoxin findings to all sepsis patients and should be considered when interpreting the results.

### 3.4. Sepsis Analysis

A comprehensive analysis of the patients' demographic and clinical characteristics demonstrated statistically significant differences between patients with and without sepsis in terms of the presence of an underlying oncologic pathology, positive blood culture findings, levels of CRP and PCT, time to death, and length of stay in the intensive care unit. Additionally, logistic regression analyses conducted to identify risk factors for sepsis revealed that positive blood culture results and length of stay in the intensive care unit emerged as independent risk factors (Table 3). The ROC analysis performed to determine diagnostic accuracy of both individual and combined clinical parameters for sepsis prediction. Also, cut-off values were determined (Table 4).

### 3.5. Prognosis Analysis

A detailed analysis of patients' demographic and clinical profiles revealed statistically significant differences between deceased and discharged patients regarding the presence of underlying oncologic pathology, sepsis status, and levels of IL-6 and CRP. Additionally, logistic regression analyses performed to identify risk factors for mortality revealed that sepsis and IL-6 emerged as independent risk factors (Table 5). The ROC analysis performed to determine diagnostic accuracy of both individual and combined clinical parameters for mortality prediction. Also, cut-off values were determined (Table 6).

### 3.6. Combination Analysis

In our study, in addition to individual measurements, we evaluated the sensitivity and specificity of various biomarkers when used in combination through ROC analysis. When used together, IL-6 and CRP demonstrated 67% sensitivity and 62% specificity in predicting sepsis. Furthermore, when the presence of bacteremia was included as an additional criterion, the IL-6 and CRP combination reached a sensitivity of 84%; however, the specificity remained considerably low at 34%. When used together, PCT and CRP demonstrated 85% sensitivity and 80% specificity in predicting sepsis. Also, when the presence of bacteremia was included as an additional criterion, the PCT and CRP combination reached a sensitivity of 90% and the specificity of 80% with considerably high AUC at 0.88 for prediction of sepsis. 

In the prediction of mortality, the combination of IL-6 and CRP reached 92% sensitivity in the presence of bacteremia but specificity was relatively low at 78%. IL6 and CRP together demonstrated 83% sensitivity, 80% specificity, and a high diagnostic accuracy with an AUC of 0.90 (*p* < 0.001) in cases where no bacteria were detected in blood cultures. Also, IL-6, PCT and CRP combination demonstrated 90% sensitivity and 85% specificity for predicting mortality with bacteremia.

## 4. Discussion

Sepsis is a critical challenge in clinical medicine and early and precise diagnosis, and therapy guidance are critical for better patient outcomes. In this context, biomarkers are being investigated and implemented into clinical practice to improve diagnostic accuracy and guide treatment decisions [23]. Among the hundreds of biomarkers, CRP and PCT are the most thoroughly investigated and routinely used biomarkers [17]. PCT is produced by almost all organs and activated macrophages in response to inflammatory stimuli, with serum levels rising within 3–4 h and peaking around 24 h [24]. CRP is an acute-phase protein produced solely by the liver in response to proinflammatory cytokines (most notably interleukin 6), with serum levels rising within 4–6 h of stimulation, double every 8 h [25]. IL-6 promotes T and B cell proliferation and differentiation while also stimulating the synthesis and release of acute-phase proteins, with peak levels occurring within 2 h of an inflammatory response. IL-6 responds faster to infections than CRP and PCT, cementing its status as a key early indicator for sepsis [15]. In theory, these biomarkers are peaking at roughly 24 h, offering a reasonable temporal window for detection during early sepsis. Furthermore, considering that blood culture systems typically yield a positive signal within 24 h when growth is present, the blood sample collected at the initial admission to intensive care holds significant value for both diagnostic and prognostic purposes.

CRP is a useful biomarker for the early detection of sepsis, offering high sensitivity but limited specificity [23]. Although many studies regard PCT as superior to CRP, it is not a definitive test for diagnosing sepsis, as elevated PCT levels can also occur in other conditions [26,27,28,29]. Nevertheless, both markers demonstrate limited diagnostic performance when used alone, and their primary value may lie in ruling out sepsis rather than confirming it [29,30]. IL-6 independently predicts sepsis with moderate sensitivity and relatively high specificity, supporting its potential as a complementary marker in early diagnosis [15,31]. Moreover, when combined with PCT, IL-6 has been shown to correlate with the severity and prognosis of sepsis, indicating its usefulness in tracking disease progression [32]. Simultaneous measurement of multiple biomarkers may help address the limitations of relying on a single marker. Combining biomarkers that reflect different pathways involved in sepsis could be especially advantageous [17,23]. Additionally, we recommend including the presence or absence of bacteremia in the combinations of biomarker sets quantitatively measured by analyzers. We obtained sensitivity results for singular and combination of biomarkers comparable to those reported in previous studies and meta-analyses [15,30,31,33,34]. In our study, the CRP + PCT combination emerged as the best predictor for sepsis, demonstrating 85% sensitivity, 80% specificity, and an AUC of 0.85. However, when the presence of bacteremia, regardless of the bacterial species isolated, was incorporated as an additional criterion, the CRP + PCT combination achieved an improved performance, with 90% sensitivity, 80% specificity, and an AUC of 0.88. Similarly, in prognostic prediction, the CRP + PCT + IL-6 combination identified in our study demonstrated acceptable performance, with a sensitivity of 75% and a specificity of 77%. Notably, in patients with bacteremia, the same combination showed enhanced predictive power, achieving 90% sensitivity and 85% specificity. In our study, SAA and endotoxin were not found to be useful for either the diagnosis of sepsis or the prediction of prognosis.

The clinical scoring systems such as qSOFA, SIRS, and NEWS are widely utilized in sepsis diagnosis, their limited sensitivity and specificity highlight the need for complementary biomarker support, particularly in cases lacking clear organ dysfunction [35]. Incorporating multiple biomarkers that capture distinct aspects of sepsis pathophysiology such as inflammatory, immune, and metabolic responses represent a promising approach to enhancing diagnostic accuracy [23]. Furthermore, integrating machine learning with real-time biomarker and physiological data presents a forward-looking strategy for sepsis prediction, with the potential to facilitate earlier intervention in high-risk patients [36]. As this study was conducted in a tertiary referral ICU, the patient cohort consisted predominantly of severely ill individuals, including those transferred from other wards or external hospitals. This setting limited our ability to reliably determine admission causes, distinguish between community and hospital-acquired infections, or comprehensively assess frailty status. In addition, the high prevalence of multiple comorbidities in this population complicated stratification, and no significant relationship was observed between comorbidity burden and pathogen distribution. These factors should be considered when generalizing our findings to other ICU settings.

## 5. Conclusions

In conclusion, no single biomarker provides perfect diagnostic accuracy. However, combining markers such as PCT, CRP, and IL-6 with clinical judgment and blood culture results may enhance early sepsis detection. Due to the 48–72-hour timeframe required for the identification of the growing microorganism and antibiotic susceptibility results; blood culture may not be considered among the primary tests for early sepsis diagnosis. However, our study demonstrated that the presence of bacteremia in the initial blood sample taken from intensive care patients holds significant diagnostic and prognostic value, even without waiting for species-level identification. Advanced computational approaches, including machine learning algorithms, could further improve the integration of multiple biomarkers and clinical variables in sepsis diagnosis and prognosis.

## Figures and Tables

**Table 1:** Demographic characteristics and clinical parameters of the patients.

Parameters	
Age (x ± sd)	65.79 ± 15.14
Sex (*n*, %)	
Female	52 (39.3%)
Male	80 (60.6%)
Primary Pathology (*n*, %)	
Oncologic Diseases	56 (42.4%)
Cardiovascular and Respiratory Diseases	53 (40.2%)
Gastrointestinal and Hepatic Diseases	11 (8.3%)
Other	12 (9.1%)
Sepsis (*n*, %)	
Present	100 (75.8%)
Absent	32 (24.2%)
Prognosis (*n*, %)	
Ex	98 (74.2%)
Discharge	34 (25.8%)
Blood Culture (*n*, %)	
Positive	69 (52.3%)
Negative	63 (47.7%)
IL-6 (pg/mL) (x ± sd)	714.22 ± 1420.26
CRP (ng/mL) (x ± sd)	72,371.59 ± 36,361.11
PCT (ng/mL) (x ± sd)	7.07 ± 21.04
SAA (µg/mL) (x ± sd)	206.51 ± 120.70
Endotoxin (pg/mL) (x ± sd)	61.90 ± 32.62
Time to Mortality (days) (x ± sd)	13.88 ± 14.62
ICU Length of Stay (days) (x ± sd)	14.69 ± 14.17

**Table 2:** Species-level classification of bacteria isolated in blood cultures (Group II).

Microorganism	*n* (%)
*Klebsiella pneumoniae*	18 (26.0)
*Candida albicans*	8 (11.5)
Coagulase-negative *Staphylococci*	8 (11.5)
*Enterococcus faecium*	7 (10.1)
*Acinetobacter baumannii*	6 (8.7)
*Staphylococcus aureus*	6 (8.7)
*Enterococcus fecalis*	4 (5.8)
*Escherihia coli*	4 (5.8)
*Pseudomonas aureginosa*	3 (4.3)
Other	5 (7.2)
Total	69 (100)

**Table 3:** Results of Univariate and Multivariate Analyses for Determining Factors of Sepsis.

	Sepsis			
	Univariate ^1^		Multivariate ^1^	
Parameters	OR (%95 CI)	*p* ^2^	OR (%95 CI)	*p* ^2^
Age	1.00 (0.97–1.03)	0.804		
Sex				
Female	1	-		
Male	1.07 (0.47–2.41)	0.870		
Blood Culture				
Positive	1	-	1	-
Negative	7.37 (2.78–19.58)	0.001	13.95 (3.72–52.32)	0.001
IL-6	1.48 (0.87–2.51)	0.145		
CRP	4.25 (2.31–7.82)	0.001	3.80 (1.95–7.40)	0.001
PCT	3.89 (2.10–7.20)	0.001	3.25 (1.70–6.22)	0.001
SAA	1.27 (0.53–3.04)	0.589		
Endotoxin	0.99 (0.98–1.00)	0.474		
Time to mortality	1.19 (1.00–1.41)	0.047		
ICU Length of Stay	1.05 (1.00–1.09)	0.022	61.97 (7.19–533.5)	0.001

^1^ Logistic Regression Analysis, Univariate and Multivariate Results, *R^2^*: 0.60. ^2^ *p*-values < 0.05 were considered statistically significant.

**Table 4:** ROC Analysis of Clinical Parameters for the Diagnosis of Sepsis.

Parameters	Cut-Off	Sensitivity (%)	Specificity (%)	AUC (%95 CL)	*p* Value ^1^
Age	69.5	49	47	0.50	0.933
IL-6 (pg/mL)	808	63	62	0.62	0.044
CRP (ng/mL)	831	82	75	0.81	0.006
PCT (ng/mL)	1.8	78	70	0.79	0.001
Endotoxin (pg/mL)	63.6	41	41	0.41	0.122
ICU Length of stay	6.5	70	50	0.63	0.013
IL-6 * CRP	-	67	62	0.63	0.034
IL-6 * Age	-	63	62	0.63	0.047
IL-6 * Endotoxin	-	60	60	0.60	0.77
Endotoxin * ICU length of stay	-	60	50	0.59	0.105
ICU Length of stay * Age	-	64	60	0.64	0.006
ICU Length of stay * IL-6	-	72	60	0.66	0.006
ICU Length of stay * CRP	-	64	63	0.67	0.001
CRP * PCT	-	85	80	0.85	0.001

^1^ Hanley & McNeil method, *p*-values < 0.05 were considered statistically significant; CL: Confidence Limit; *: Combination analysis.

**Table 5:** Results of Univariate and Multivariate Analyses for Prognostic Factors.

	Prognosis			
	Univariate ^1^		Multivariate ^1^	
Parameters	OR (%95 CI)	*p* ^2^	OR (%95 CI)	*p* ^2^
Age	1.00 (0.98–1.03)	0.644		
Sex				
Female	1	-		
Male	0.93 (0.42–2.08)	0.873		
Sepsis				
Absent	1	-	1	-
Present	10.23 (4.11–25.47)	0.001	60.26 (11.17–324.90)	0.001
Blood culture				
Negative	1	-	1	-
Positive	1.82 (0.82–4.03)	0.135	0.36 (0.10–1.26)	0.112
IL-6	4.48 (2.17–9.21)	0.001	4.123 (1.86–9.11)	0.001
CRP	1.59 (0.99–2.56)	0.054		
PCT	1.36 (0.57–3.27)	0.480		
SAA	1.00 (0.99–1.00)	0.205		
Endotoxin	1.31 (0.42–4.02)	0.633		
ICU Length of Stay	0.29 (0.100–0.88)	0.029	0.036 (0.005–0.268)	0.857

^1^ Logistic Regression Analysis, Univariate and Multivariate Results *R^2^*: 0.60. ^2^ *p*-values < 0.05 were considered statistically significant.

**Table 6:** ROC Analysis of Clinical Parameters for Prognosis.

Parameters	Cut-Off	Sensitivity (%)	Specificity (%)	AUC (%95 CL)	*p* Value ^1^
Age	69.5	49	47	0.49	0.864
IL-6 (pg/mL)	639	74	73	0.78	0.000
CRP (ng/mL)	817	68	65	0.68	0.001
Endotoxin (pg/mL)	513	53	41	0.243	0.187
ICU Length of Stay	9.5	43	33	0.36	0.005
IL-6*CRP	-	74	70	0.77	<0.001
IL-6 * ICU Length of Stay	-	67	65	0.70	<0.001
CRP * ICU Length of Stay	-	53	50	0.54	0.550
Endotoxin * ICU Length of Stay	-	39	35	0.36	0.008
CRP * PCT	-	73	75	0.76	<0.001
CRP * PCT * IL-6	-	75	77	0.78	0.001

^1^ Hanley & McNeil method, *p*-values < 0.05 were considered statistically significant; CL: Confidence Limit; *: Combination analysis.

## Data Availability

The data that support the findings of this study are available from the corresponding author upon reasonable request.

## Supplementary Materials

The following supporting information can be downloaded at: https://www.mdpi.com/article/10.3390/pathogens14090907/s1, Table S1: Bonferroni-adjusted *p*-values for univariate logistic regression (corresponding to Table 3); Table S2: ROC analysis with Bonferroni-adjusted *p*-values (corresponding to Table 4); Table S3: Univariate logistic regression results with Bonferroni-adjusted *p*-values (corresponding to Table 5); Table S4: ROC analysis with Bonferroni-adjusted *p*-values (corresponding to Table 6).

## Abbreviations

The following abbreviations are used in this manuscript:

ICU	Intensive care unit
BSI	Bloodstream infection
EMB	Eosin Methylene Blue
CRP	C-Reactive protein
IL-6	Interleukin-6
PCT	Procalcitonin
SAA	Serum amyloid A
CLIA	Chemiluminescence immunoassay
ELISA	Enzyme-linked Immunosorbent Assay
SIRS	Systemic inflammatory response syndrome
