# Supplementary material for: First Signs at Admission: Prognostic Value of Initial Proinflammatory Markers for Sepsis and Mortality in ICU Patients
Source: Pathogens. 2025 Sep 10;14(9):907. doi: 10.3390/pathogens14090907 (PMC12472384; doi:10.3390/pathogens14090907)
Supplement: Supplementary file 1 [file pathogens-14-00907-s001.zip › pathogens-3829286-supplementary.pdf]

Supplementary Table S1. Bonferroni-adjusted p-values for univariate logistic regression (corresponding to Table 3).

	Sepsis				
	Univariate[1]		Multivariate[1]		
Parameters	OR (%95 CI)	*P*[2]	OR (%95 CI)	*P*[2]	*Univariate Bonferroni-adj P value*
Age	1.00 (0.97-1.03)	0.804			1.00
Sex					
Female	1	-			
Male	1.07 (0.47-2.41)	0.870			1.00
Blood Culture					
Positive	1	-	1	-	
Negative	7.37 (2.78-19.58)	0.001	13.95 (3.72-52.32)	0.001	**0.010**
IL-6	1.48 (0.87-2.51)	0.145			1.00
CRP	4.25 (2.31-7.82)	0.001	3.80 (1.95-7.40)	0.001	**0.010**
PCT	3.89 (2.10-7.20)	0.001	3.25 (1.70-6.22)	0.001	**0.010**
SAA	1.27 (0.53-3.04)	0.589			1.00
Endotoxin	0.99 (0.98-1.00)	0.474			1.00
Time to mortality	1.19 (1.00-1.41)	0.047			0.475
ICU Length of Stay	1.05 (1.00-1.09)	0.022	61.97(7.19-533.5)	0.001	0.222

[1] *Logistic Regression Analysis, Univariate & Multivariate Results R2:0.60*
[2] *P*-values <0.05 were considered statistically significant.

Supplementary Table S2. ROC analysis with Bonferroni-adjusted p-values (corresponding to Table 4).

**Parameters**	**Cut-off**	**Sensitivity (%)**	**Specifity (%)**	**AUC (%95 CL)**	***P* value[1]**	***Bonferroni-adj P value***
Age	69.5	49	47	0.50	0.933	1.000
IL-6 (pg/mL)	808	63	62	0.62	0.044	0.616
CRP (ng/mL)	831	82	75	0.81	0.006	0.084
PCT (ng/mL)	1.8	78	70	0.79	0.001	0.014
Endotoxin (pg/mL)	63.6	41	41	0.41	0.122	1.000
ICU Length of stay	6.5	70	50	0.63	0.013	0.182
IL-6*CRP	-	67	62	0.63	0.034	0.476

IL-6*Age	-	63	62	0.63	0.047	0.658
IL-6*Endotoxin	-	60	60	0.60	0.77	1.000
Endotoxin*ICU length of stay	-	60	50	0.59	0.105	1.000
ICU Length of stay *Age	-	64	60	0.64	0.006	0.084
ICU Length of stay *IL-6	-	72	60	0.66	0.006	0.084
ICU Length of stay *CRP	-	64	63	0.67	0.001	**0.014**
CRP*PCT	-	85	80	0.85	0.001	**0.014**

[1] *P*-values <0.05 were considered statistically significant.

Supplementary Table S3. Univariate logistic regression results with Bonferroni-adjusted p-values (corresponding to Table 5).

	**Prognosis**				
	**Univariate[1]**		**Multivariate[1]**		***Univariate Bonferroni-adj P value***
Parameters	OR (%95 CI)	*P*[2]	OR (%95 CI)	*P*[2]	
Age	1.00 (0.98-1.03)	0.644			1.000
Sex					
Female	1	-			
Male	0.93 (0.42-2.08)	0.873			1.000
Sepsis					
Absent	1	-	1	-	
Present	10.23 (4.11-25.47)	0.001	60.26 (11.17-324.90)	0.001	**0.010**
Blood culture					
Negative	1	-	1	-	
Positive	1.82 (0.82-4.03)	0.135	0.36 (0.10-1.26)	0.112	1.000
IL-6	4.48 (2.17-9.21)	0.001	4.123 (1.86-9.11)	0.001	**0.010**
CRP	1.59 (0.99-2.56)	0.054			0.540
PCT	1.36 (0.57-3.27)	0.480			1.000
SAA	1.00 (0.99-1.00)	0.205			1.000
Endotoxin	1.31 (0.42-4.02)	0.633			1.000
ICU Length of Stay	0.29 (0.100-0.88)	0.029	0.036 (0.005-0.268)	0.857	0.290

[1] *Logistic Regression Analysis, Univariate & Multivariate Results R2:0.60*

[2] *P*-values <0.05 were considered statistically significant.

Supplementary Table S4. ROC analysis with Bonferroni-adjusted p-values (corresponding to Table 6).

**Parameters**	**Cut-off**	**Sensitivity (%)**	**Specifity (%)**	**AUC (%95 CL)**	***P* value[1]**	***Bonferroni-adj P value***
Age	69.5	49	47	0.49	0.864	1.000
IL-6 (pg/mL)	639	74	73	0.78	0.000	**0.000**
CRP (ng/mL)	817	68	65	0.68	0.001	**0.011**
Endotoxin (pg/mL)	513	53	41	0.243	0.187	1.000
ICU Length of Stay	9.5	43	33	0.36	0.005	0.055
IL-6*CRP	-	74	70	0.77	< 0.001	**0.011**
IL-6* ICU Length of Stay	-	67	65	0.70	< 0.001	**0.011**
CRP* ICU Length of Stay	-	53	50	0.54	0.550	1.000
Endotoxin* ICU Length of Stay	-	39	35	0.36	0.008	0.088
CRP* PCT	-	73	75	0.76	< 0.001	**0.011**
CRP* PCT* IL-6	-	75	77	0.78	0.001	**0.011**

[1] *P*-values <0.05 were considered statistically significant.
